# Breast metastasis of ilial carcinoid tumor: Case report and literature review

**DOI:** 10.1186/1477-7819-4-15

**Published:** 2006-03-27

**Authors:** Chakshu Gupta, Ashok K Malani, Sandeep Rangineni

**Affiliations:** 1 Department of Oncology, Heartland Regional Medical Center, St. Joseph, Missouri 64506, USA; 2 Department of Pathology, Heartland Regional Medical Center, St. Joseph, Missouri 64506, USA

## Abstract

**Background:**

Metastatic breast carcinoids are rare neoplasms. They can be mistaken for primary breast carcinoma both clinically and radiologically, even with known history of carcinoid tumor elsewhere in the body.

**Case presentation:**

We report a case of unilateral breast metastasis from carcinoid tumor of the small intestine in a 52-year-old woman who was successfully treated by lumpectomy and radiation therapy. An extensive review of the literature reveals only a few cases of metastatic carcinoid to the breast from small intestinal primaries.

**Conclusion:**

Clinical suspicion for metastasis should be high in a patient with breast mass and history of known carcinoid elsewhere in the body. Lumpectomy alone may be effective in these patients. Mastectomy and especially axillary dissection could be avoided. Their histological appearance may mimic ductal adenocarcinoma of the breast. However, the distinction is important due to differences in management and prognosis.

## Background

Metastatic tumors to breast represent a mere 1–2% of all breast tumors [1,2]. Common tumors that metastasize to the breast include those from the lung, prostate, thyroid, kidney, hematopoietic system, and malignant melanoma [3-5]. In children, rhabdomyosarcoma is reported to be the most common primary source of metastatic breast lesions [6]. In comparison, metastatic neoplasms from gastrointestinal primaries are rare and include the stomach, pancreas, esophagus, and colon. The majority of these tumors are adenocarcinomas [6]. Small intestinal carcinoids metastasizing to the breast have only sporadically been reported in the literature [7]. Metastatic breast carcinoid can be easily mistaken for primary breast carcinoma. This may potentially be detrimental for the patient, especially if the primary surgery is a mastectomy with axillary lymph node dissection. Many times a primary lesion may not be discovered and breast metastasis may be the presenting feature of an occult carcinoid [8,9]. Primary carcinoid tumors of the breast are also reported in the literature, although this view is no longer accepted [7,10,11]. We report a case of metastatic breast carcinoid in a 52-year-old postmenopausal nun along with a review the literature.

## Case presentation

A 52-year-old postmenopausal woman, a Nun by profession, presented with complaints of alternating diarrhea and constipation, and abdominal distension of several days duration. Her medical history was significant for hypertension, diabetes mellitus, and psoriasis. Physical examination revealed a palpable abdominal mass extending from the pelvis to above the umbilicus. It was firm in consistency and very suspicious for malignancy. Subsequent evaluation for serum tumor markers revealed an elevated CA125 (1017, reference range < 35). Computed tomography (CT) scan of the abdomen showed a large solid midline mass in the pelvis and lower abdomen, extending to above the umbilicus and measuring up to 19 cm. It had multiple areas of low attenuation within it. In addition, several hyperdense lesions were noted in the liver, measuring approximately 1–2 cm in diameter. A third lesion was present in the posterior part of the pelvis, was solid appearing, and possibly involving the right ovary. It measured 10 cm and was noted to be pressing on the inferior vena cava and the right lower ureter causing mild hydronephrosis.

**Figure 1:**
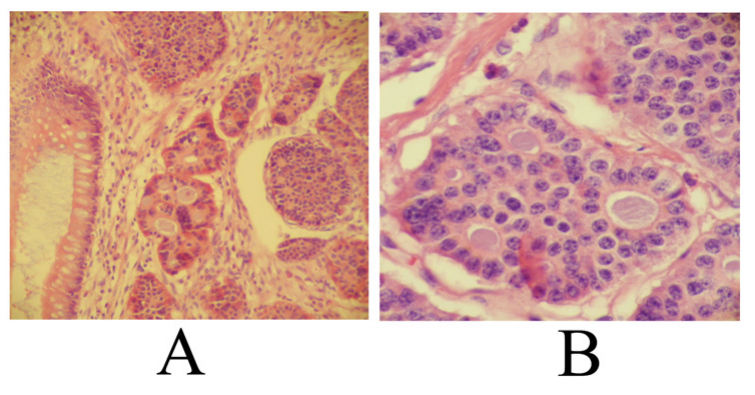
**1A **Infiltrating nests of tumor within small intestinal wall. Normal intestinal epithelium is at the left of the image (H&E, 100×). **1B**. The cells contain round to oval nuclei with vesicular chromatin (H&E, 400×)

She underwent a segmental resection of the ileum, along with a total abdominal hysterectomy, bilateral salphingo-ophorectomy, and intraoperative needle biopsies of the liver masses. Histological examination revealed a large carcinoid in the ileum with metastasis to regional lymph nodes. The tumor was present as nests and sheets of uniform cells with focal gland formation (figure 1A). Cells contained modest amount of eosinophilic cytoplasm and monotonous appearing nuclei with vesicular nucleoli (figure 1B). Focal areas of mucosal ulceration, as well as extensive perineural and vascular invasion were present. Metastatic deposits of carcinoid tumor were also found in both ovaries and in the liver.

A 24-hour urine specimen revealed predictably high levels of 5-HIAA (5 Hydroxy Indole Acetic acid) at 45.2 (reference range 0.5–9 mg/24 hours). The patient was followed regularly with 24-hour urine 5-HIAA levels and CT scans of abdomen and chest. Two years post diagnosis; mild progression of her liver disease was noted. The patient was initiated on subcutaneous octreotide (Sandostatin) at a dose of 50 mg/m^2 ^twice daily.

**Figure 2:**
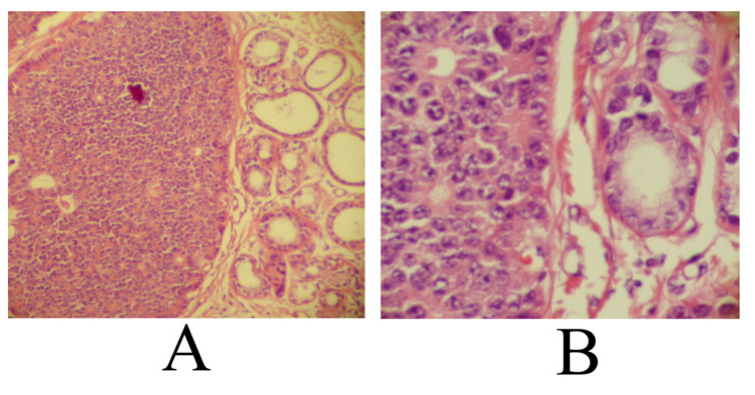
**2**A). Large nest of tumor infiltrating within breast parenchyma. Normal ducts are seen at the right of the image (H&E, 100×). 2B). The histology of the tumor (left half of image) is similar to that from the intestine. The malignant cells contain round to oval nuclei with vesicular chromatin, rare mitosis, and no necrosis (H&E, 400×).

She remained stable for three and one half years, when she discovered a lump in her left breast. Clinical examination revealed a firm mobile mass in the breast suspicious for tumor. Mammograms were significant for an irregular, marginated mass of 2.2 × 2.0 cm in the upper inner quadrant of the left breast. An ultrasound confirmed these findings, revealing a hypoechoic, irregularly marginated mass highly suspicious for malignancy. The patient underwent lumpectomy of the breast mass for a definitive diagnosis. The gross specimen had a tan grey firm nodule of 2.5 × 1.5 × 1.5 cm with smooth borders within otherwise unremarkable fatty tissue. Histological examination revealed a 2.5 cm nodule containing infiltrating cords and nests of cells morphologically similar to her original ileal carcinoid tumor. The larger nests demonstrated an acinar pattern with rosette formation. The cells contained round to oval nuclei with a fine reticular chromatin pattern (Figure 2A and 2B). Necrosis was absent and mitoses were rare. Focally margins were positive for tumor. A partial mastectomy was performed to remove residual tumor. The patient received adjuvant radiation therapy to the affected breast. She has had no recurrences and has had stable liver disease (metastatic carcinoid) for 5 years following her breast surgery.

## Discussion

Carcinoids are slow growing neoplasms derived from enterochromaffin cells and are thus neuroendocrine in nature. They arise most commonly in the gastrointestinal or respiratory tract [12]. Carcinoid syndrome occurs in approximately 5% of patients with intestinal carcinoids and manifests as episodes of diarrhea, abdominal pain, and flushing [13]. They typically occur in the presence of hepatic metastasis because the liver can no longer metabolize the polypeptides (including serotonin and substance P) produced by the tumor cells. This phenomenon is only observed in intestinal carcinoids due to the venous drainage of this organ system. When the primary tumor is extraintestinal, carcinoid syndrome may be produced without hepatic metastasis [8].

Carcinoid tumors are now considered at the well-differentiated end of the spectrum of neuroendocrine carcinomas. By definition, typical carcinoids have a bland morphology, lack necrosis, and have less 2 mitoses per 10 high power fields. However, carcinoids are malignant neoplasms and retain the capacity to metastasize. Morphological features of neuroendocrine carcinomas do not predict their metastatic potential [7]. Common sites of metastasis are to the lungs, liver, and the peritoneum. Metastases are least likely to involve bone, skin, and brain [12].

The first case of metastatic breast carcinoid appeared in the literature in 1957 and was an autopsy finding [4]. In a review of literature by Rubio *et al*, the ileum was found to be the most common primary site for metastatic breast carcinoid. The appendix, duodenum, pancreas, lungs, and ovaries were other primary sites, occurring with equal frequency in their review [8]. Metastatic breast carcinoids may present clinically as single or multiple well-circumscribed lesions, with a firm consistency. In several published reports, they were clinically interpreted as fibroadenomas, or uncommonly as medullary or mucinous of ductal carcinoma [4,14,15]. In a review by Fishman *et al*, 8 of 13 (61.5%) patients with metastatic breast carcinoid were initially considered to have primary breast carcinoma and were subjected to mastectomies [16]. The diagnosis of metastatic breast carcinoid was made after review of the histology of the mastectomy specimen after it was discovered that the patient had a primary carcinoid at a different site. In our patient, clinical suspicion of metastatic carcinoid was high, although a primary breast carcinoma was also in the differential diagnosis. Misdiagnosis of breast carcinoid as primary ductal carcinoma has also been reported even with a prior history of carcinoid tumor at a different site in the patient [16]. Organoid nests of cells with rosette-like structures can mimic the pattern of solid or cribriform DCIS (ductal carcinoma in-situ).

Argentaffin and argyrophil stains are usually positive in carcinoid tumors of the small bowel, whereas "primary" carcinoid tumors of the breast demonstrate only argyrophilic granules [12]. Recently, the very existence of primary breast carcinoids has been discredited [7]. Carcinoid tumors metastatic to the breast can show estrogen receptor positivity creating problems in differential diagnosis with a primary ductal carcinoma of the breast [18]. Clinically, there are no reliable criteria to distinguish metastatic breast carcinoid from primary breast carcinoid. In-situ ductal carcinoma may be the only absolute proof of primary nature of breast carcinoma [4]. Immunohistochemical and ultra structural analysis can be extremely useful in the accurate recognition of neuroendocrine nature of these tumors. These include synaptophysin, chromogranin A and B, and neuron specific enolase (NSE). More recent immunostains include prohormone convertase (PC3), CDX-2, and peptidylglycine a-amidating monooxygenase (PGM).

A modified radical mastectomy was performed in the majority of cases reported in the literature, as the clinical diagnosis was that of a primary breast carcinoma. Lumpectomy has been performed in only a few cases [6,7,13,19]. Large series of such tumors are lacking in the literature and we cannot recommend a more specific management approach. Large series of such tumors are lacking in the literature and we cannot recommend a more specific management approach, however, axillary lymph node dissection may not be necessary. Estrogen receptors may be positive in carcinoid tumors, however, none have been reported positive in metastatic breast carcinoids to our knowledge. HER-2/neu may be positive and has been described in at least one report of metastatic carcinoid to the breast [7]. Role of chemotherapy and radiotherapy is also unclear.

In conclusion, metastatic carcinoid tumors to the breast can mimic primary breast carcinoma. In patients with a breast mass and a known history of carcinoid tumor, one should have a high index of suspicion for metastatic disease. A lumpectomy should be offered to these patients rather than modified radical mastectomy as first line therapy.

## Competing interests

The author(s) declare that they have no competing interests.

## Authors' contributions

**AKM **was involved in clinical management of the patient following her surgery. **CG **reviewed the pathology of the patient. **SR **was also involved in the post-operative oncologic management of the patient. All three were involved in review of radiology films, medical records, literature search, manuscript preparation and critical review of final manuscript. All authors approved the final version of the manuscript.
